# Investigating How Physical Activity Intensity Relates to Restless Sleep: A Case Study Using a Smart Ring

**DOI:** 10.1093/geroni/igaf122.3614

**Published:** 2025-12-31

**Authors:** Kangeun Lee, Jungjoo Lee, Junhyoung Kim

**Affiliations:** Indiana University, College Station, Texas, United States; Texas A&M University, Texas A&M University, Texas, United States; Texas A&M University, College Station, Texas, United States

## Abstract

**Background:**

Older adults with mild cognitive impairment (MCI) often experience restless sleep. Physical activity is a coping strategy to reduce such disturbances, but self-reported data are limited by recall bias and cognitive difficulties.

**Aim:**

This study examined the longitudinal relationship between restless sleep and physical activity intensity in older adults with MCI using continuous monitoring with a smart ring system.

**Method:**

Seven older adults with MCI were monitored using Oura smart rings for 14 days. The rings, equipped with photoplethysmography, accelerometer, and gyroscope sensors, tracked changes in movement and heart rate to identify sleep stages and periods of restlessness. Physical activity intensity was computed from heart rate variability and skin temperature, and classified as light, moderate, or vigorous using the Metabolic Equivalent of Task. Generalized estimating equations were applied to account for the small sample size.

**Results:**

Vigorous activity was significantly associated with reduced restlessness, with each additional second in activity leading to a 0.18-second decrease in restlessness (B = -0.18, SD = 0.05, p < .01, 95% CI [-0.29, -0.07]). Moderate activity showed a small, non-significant positive association (B = 0.01, SD = 0.01, 95% CI [-0.01, 0.03]). Light activity showed a significant but slight reduction in restlessness with more time in light activity (B = -0.01, SD = 0.01, p < .01, 95% CI [-0.01, -0.01]).

**Conclusion:**

Vigorous and light physical activity were significantly linked to reduced restlessness during sleep, whereas moderate activity showed no meaningful effect. Further studies should examine potential confounding factors affecting moderate activity outcomes.

